# Supplementary material for: A Polarizable CASSCF/MM Approach Using the Interface Between OpenMMPol Library and Cfour
Source: J Comput Chem. 2024 Dec 24;46(1):e27550. doi: 10.1002/jcc.27550 (PMC11668234; doi:10.1002/jcc.27550)
Supplement: Supplementary file 1 — Data S1 Supporting Information. [file JCC-46-0-s001.pdf]

Supporting Information for: A polarizable CASSCF/MM approach using the interface between OpenMMPol library and CFour

Tommaso Nottoli[1], Mattia Bondanza[1], Filippo Lipparini[1] and Benedetta Mennucci[1]

[1]Dipartimento di Chimica e Chimica Industriale, Università di Pisa, Via G. Moruzzi 13, I-56124 Pisa, Italy

S1. Additional calculations for Dronpa system

We report in the following tables further results obtained using different active spaces for the CASSCF method. In particular we show in Table S1 the bond length alternation (BLA) parameter, in Table S2 the hydrogen-bond lengths, and in Table S3 the value of the dihedral angle formed by the carbon atoms connecting the benzene moiety to the imidazole ring of Dronpa's chromophore.

Table S1. BLA values (in Å) for Dronpa's chromophore computed with different CAS active spaces computed in vacuum and in the protein with the two different force fields.

Method	Vacuum	Amber	AMOEBA
CAS(4,4)	0.027	0.002	-0.018
CAS(8,8)	0.037	-0.004	-0.017
CAS(10,10)	0.048	-0.004	-0.020
CAS(12,12)	0.041	0.010	-0.020

Table S2. Hydrogen bond lengths between the two oxygen atoms of Dronpa's chromophore and the residues/water. All values are in Å.

	**Ser**		**Wat**		**Arg_1**		**Arg_2**		**Arg_3**	
**Method**	Amber	AMOEBA	Amber	AMOEBA	Amber	AMOEBA	Amber	AMOEBA	Amber	AMOEBA
CAS(4,4)	1.743	1.790	1.740	1.834	1.886	1.950	2.459	2.504	1.781	1.926
CAS(8,8)	1.741	1.785	1.738	1.827	1.909	1.952	2.450	2.506	1.795	1.930
CAS(10,10)	1.740	1.788	1.736	1.830	1.909	1.969	2.449	2.509	1.795	1.951
CAS(12,12)	1.751	1.787	1.742	1.828	1.898	1.970	2.455	2.508	1.787	1.952

Table S3. Dihedral angle in degrees for Dronpa's chromophore.

Method	Vacuum	Amber	AMOEBA
CAS(4,4)	180	175	174
CAS(8,8)	180	175	174
CAS(10,10)	180	175	173
CAS(12,12)	180	174	173

S2. Additional calculations for OCP system

Additional data is provided also for the OCP system. In the tables below, we present results obtained using various CAS active spaces for the geometrical parameters investigated in the main paper. Specifically, we report in Table S4 the BLA, in Table S6 the hydrogen-bond lengths, in Table S5 the angle form between the oxygen of CAN and the two residues.

Table S4. BLA values (in Å) for OCP's chromophore computed with different CAS active spaces computed in vacuum and in the protein with the two force fields.

Method	Vacuum	Amber	AMOEBA
CAS(8,8)	0.157	0.149	0.152
CAS(8,10)	0.157	0.150	0.149
CAS(8,12)	0.151	0.147	0.149
CAS(10,12)	0.141	0.143	0.142
CAS(12,12)	0.155	0.136	0.139
CAS(12,14)	0.149	0.154	0.134

Table S5. Angle between the oxygen atom of CAN and the two residues (Trp-288 and Tyr-201).

Method	Amber	AMOEBA
CAS(8,8)	90	87
CAS(8,10)	90	87
CAS(8,12)	90	87
CAS(10,12)	90	87
CAS(12,12)	90	87
CAS(12,14)	90	87

Table S6. Hydrogen bond lengths. All values are in Å.

	**Trp-288**		**Tyr-201**	
**Method**	Amber	AMOEBA	Amber	AMOEBA
CAS(8,8)	1.878	1.924	1.864	1.921
CAS(8,10)	1.878	1.925	1.864	1.924
CAS(8,12)	1.879	1.925	1.869	1.924
CAS(10,12)	1.879	1.925	1.870	1.926
CAS(12,12)	1.881	1.928	1.876	1.932
CAS(12,14)	1.877	1.928	1.859	1.934
